# 4-(4,5-Dihydro-1*H*-benzo[*g*]indazol-3-yl)pyridinium chloride dihydrate

**DOI:** 10.1107/S160053681203019X

**Published:** 2012-07-07

**Authors:** Luan-Fang Yang, Hong-Bo Hou, Yi-Ming Liu

**Affiliations:** a Department of Biology and Chemistry, Bao Shan College, Bao Shan, Yunnan 678000, People’s Republic of China

## Abstract

In the cation of the title compound, C_16_H_14_N_3_^+^·Cl^−^·2H_2_O, the cyclo­hexa-1,3-diene ring displays a screw-boat conformation and the pyridine ring is slightly twisted with respect to the pyrazole ring with a dihedral angle of 4.56 (12)°. In the crystal, ions and water mol­ecules are linked into a three-dimensional network by classical N—H⋯O, N—H⋯Cl, O—H⋯Cl and O—H⋯O hydrogen bonds and by π–π stacking inter­actions, with centroid–centroid distances of 3.7580 (14) and 3.7794 (14) Å.

## Related literature

For background to the pharmacological properties of indazole derivatives, see: Bistochi *et al.* (1981 ▶); Keppler & Hartmann (1994 ▶); Gomtsyan *et al.* (2008 ▶).
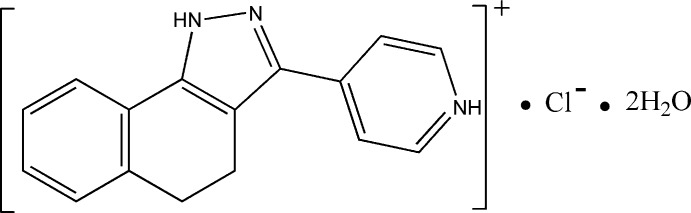


## Experimental

### 

#### Crystal data

C_16_H_14_N_3_^+^·Cl^−^·2H_2_O
*M* *_r_* = 319.78
Triclinic, 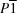

*a* = 6.7977 (5) Å
*b* = 9.4406 (7) Å
*c* = 12.2691 (9) Å
α = 93.846 (3)°
β = 96.883 (3)°
γ = 93.490 (3)°
*V* = 778.04 (10) Å^3^
*Z* = 2
Mo *K*α radiation
μ = 0.26 mm^−1^
*T* = 293 K
0.22 × 0.19 × 0.18 mm

#### Data collection

Bruker SMART CCD area-detector diffractometer
8076 measured reflections
2710 independent reflections
2185 reflections with *I* > 2σ(*I*)
*R*_int_ = 0.033

#### Refinement

*R*[*F* ^2^ > 2σ(*F* ^2^)] = 0.045
*wR*(*F* ^2^) = 0.119
*S* = 1.06
2710 reflections
199 parameters
H-atom parameters constrained
Δρ_max_ = 0.40 e Å^−3^
Δρ_min_ = −0.32 e Å^−3^

### 

Data collection: *SMART* (Bruker, 2002 ▶); cell refinement: *SAINT* (Bruker, 2002 ▶); data reduction: *SAINT*; program(s) used to solve structure: *SHELXS97* (Sheldrick, 2008 ▶); program(s) used to refine structure: *SHELXL97* (Sheldrick, 2008 ▶); molecular graphics: *ORTEP-3 for Windows* (Farrugia, 1997 ▶); software used to prepare material for publication: *WinGX* (Farrugia, 1999 ▶).

## Supplementary Material

Crystal structure: contains datablock(s) global, I. DOI: 10.1107/S160053681203019X/rz2781sup1.cif

Structure factors: contains datablock(s) I. DOI: 10.1107/S160053681203019X/rz2781Isup2.hkl

Supplementary material file. DOI: 10.1107/S160053681203019X/rz2781Isup3.cml

Additional supplementary materials:  crystallographic information; 3D view; checkCIF report

## Figures and Tables

**Table 1:** Hydrogen-bond geometry (Å, °)

*D*—H⋯*A*	*D*—H	H⋯*A*	*D*⋯*A*	*D*—H⋯*A*
N1—H1N⋯O2*W* ^i^	0.86	2.12	2.856 (2)	143
N3—H3⋯Cl1	0.86	2.32	3.1618 (19)	167
O1*W*—H1*WA*⋯Cl1^ii^	0.89	2.25	3.142 (2)	174
O1*W*—H1*WB*⋯Cl1^iii^	0.87	2.37	3.237 (3)	178
O2*W*—H2*WA*⋯Cl1	0.89	2.29	3.139 (2)	158
O2*W*—H2*WB*⋯O1*W*	0.92	1.86	2.749 (3)	162

Symmetry codes: (i) 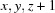; (ii) 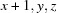; (iii) 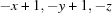.

**Fig. 1.**
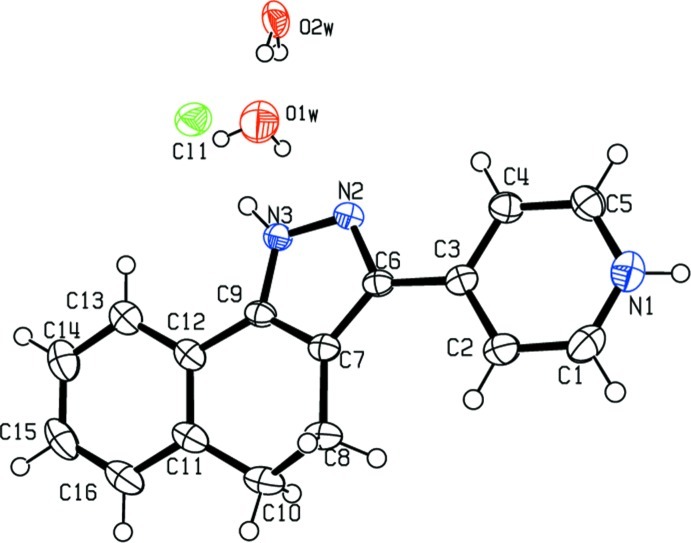
The molecular structure of title compound showing displacement ellipsoids drawn at the 50% probability level.

## supplementary crystallographic information

### Comment

Indazole derivatives exhibit a variety of pharmacological properties such as anti-inflammatory (Bistochi *et al.*, 1981), antitumor (Keppler & Hartmann, 1994), anti-HIV and analgesic properties (Gomtsyan *et al.*, 2008). Here, we present the crystal structure determination of the title compound.

The asymmetric unit of the title compound (Fig. 1) consists of an organic cation, a chloride anion a two lattice water molecules. In the cation, the cyclohexa-1,3-diene ring displays a screw-boat conformation, with atoms C8 and C10 displaced by -0.190 (3) and 0.283 (3) Å, respectively, from the C7/C9/C12/C11 mean plane, and the pyridine ring is twisted to the pyrazole ring by a dihedral angle of 4.56 (12)°. In the crystal structure, cations, anions and water molecule are linked into a three-dimensional network by classical N—H···O, N—H···Cl, O—H···Cl and O—H···O hydrogen bonds (Table 1). In addition, π–π stacking interactions [centroid-centroid distances of 3.7580 (14) and 3.7794 (14) Å] extending along the *a* axis are observed.

### Experimental

A solution of 3,4-dihydronaphthalen-1(2*H*)-one (1.46 g, 0.01 mol) was added to a stirred solution of hydrazine (0.05 g, 0.01 mol) in dry tetrahydrofuran (50 ml) at 0°C for 3 h, then n-butyllithium (0.02 mol) was added at a fast dropwise rate during a 5 min period. The solution was stirred at 0°C for an additional 30 min, then methyl isonicotinate (1.37 g, 0.01 mol) dissolved in THF (40 ml) was added to the dilithiated intermediate, and the solution was stirred for 60 min at 0°C. Finally, 20 ml of 3 *M* hydrochloric acid was added, and the two phase mixture was well stirred and heated under reflux for 45 min. The mixture was then neutralized with solid sodium bicarbonate, and the layers were separated. The aqueous layer was extracted with ether and the organic fractions were combined, evaporated, and the crude product was dissolved in hydrochloric acid (2*M*, 20 ml). The solution was filtered and the filtrate was set aside for five weeks to obtain colourless crystals.

### Refinement

C- and N-bound H atoms were placed in calculated positions and refined as riding, with C—H = 0.93–0.97 Å, N—H = 0.86 Å, and with *U*_iso_(H) = 1.2*U*_eq_(C, N). Water H atoms were located in a difference Fourier map (O—H = 0.87–0.92 Å) and refined as riding with *U*_iso_(H) = 1.5*U*_eq_(O).

### Crystal data

**Table:**

C_16_H_14_N_3_^+^·Cl^−^·2H_2_O	*Z* = 2
*M**_r_* = 319.78	*F*(000) = 336
Triclinic, *P*1	*D*_x_ = 1.365 Mg m^−^^3^
Hall symbol: -P 1	Mo *K*α radiation, λ = 0.71073 Å
*a* = 6.7977 (5) Å	Cell parameters from 2710 reflections
*b* = 9.4406 (7) Å	θ = 1.7–25.0°
*c* = 12.2691 (9) Å	µ = 0.26 mm^−^^1^
α = 93.846 (3)°	*T* = 293 K
β = 96.883 (3)°	Block, colourless
γ = 93.490 (3)°	0.22 × 0.19 × 0.18 mm
*V* = 778.04 (10) Å^3^	

### Data collection

**Table:**

Bruker SMART CCD area-detector diffractometer	2185 reflections with *I* > 2σ(*I*)
Radiation source: fine-focus sealed tube	*R*_int_ = 0.033
Graphite monochromator	θ_max_ = 25.0°, θ_min_ = 1.7°
φ and ω scans	*h* = −8→7
8076 measured reflections	*k* = −10→11
2710 independent reflections	*l* = −14→14

### Refinement

**Table:**

Refinement on *F*^2^	Primary atom site location: structure-invariant direct methods
Least-squares matrix: full	Secondary atom site location: difference Fourier map
*R*[*F*^2^ > 2σ(*F*^2^)] = 0.045	Hydrogen site location: inferred from neighbouring sites
*wR*(*F*^2^) = 0.119	H-atom parameters constrained
*S* = 1.06	*w* = 1/[σ^2^(*F*_o_^2^) + (0.0498*P*)^2^ + 0.5403*P*] where *P* = (*F*_o_^2^ + 2*F*_c_^2^)/3
2710 reflections	(Δ/σ)_max_ < 0.001
199 parameters	Δρ_max_ = 0.40 e Å^−^^3^
0 restraints	Δρ_min_ = −0.32 e Å^−^^3^

### Special details

**Table:**

Geometry. All e.s.d.'s (except the e.s.d. in the dihedral angle between two l.s. planes) are estimated using the full covariance matrix. The cell e.s.d.'s are taken into account individually in the estimation of e.s.d.'s in distances, angles and torsion angles; correlations between e.s.d.'s in cell parameters are only used when they are defined by crystal symmetry. An approximate (isotropic) treatment of cell e.s.d.'s is used for estimating e.s.d.'s involving l.s. planes.
Refinement. Refinement of *F*^2^ against ALL reflections. The weighted *R*-factor *wR* and goodness of fit *S* are based on *F*^2^, conventional *R*-factors *R* are based on *F*, with *F* set to zero for negative *F*^2^. The threshold expression of *F*^2^ > σ(*F*^2^) is used only for calculating *R*-factors(gt) *etc*. and is not relevant to the choice of reflections for refinement. *R*-factors based on *F*^2^ are statistically about twice as large as those based on *F*, and *R*-factors based on ALL data will be even larger.

### Fractional atomic coordinates and isotropic or equivalent isotropic displacement parameters (Å^2^)

**Table:**

	*x*	*y*	*z*	*U*_iso_*/*U*_eq_	
C1	0.3584 (4)	0.6698 (3)	0.8547 (2)	0.0387 (6)	
H1	0.3680	0.6302	0.9224	0.046*	
C2	0.3132 (4)	0.5827 (3)	0.75906 (19)	0.0339 (6)	
H2	0.2903	0.4850	0.7622	0.041*	
C3	0.3018 (3)	0.6421 (2)	0.65730 (17)	0.0247 (5)	
C4	0.3295 (3)	0.7899 (2)	0.65869 (18)	0.0286 (5)	
H4	0.3181	0.8331	0.5925	0.034*	
C5	0.3731 (3)	0.8721 (3)	0.75539 (19)	0.0318 (5)	
H5	0.3921	0.9705	0.7551	0.038*	
C6	0.2672 (3)	0.5559 (2)	0.55224 (17)	0.0230 (5)	
C7	0.2449 (3)	0.4070 (2)	0.52895 (17)	0.0240 (5)	
C8	0.2336 (4)	0.2806 (2)	0.5973 (2)	0.0322 (6)	
H8A	0.1014	0.2683	0.6190	0.039*	
H8B	0.3274	0.2978	0.6636	0.039*	
C9	0.2217 (3)	0.3888 (2)	0.41550 (18)	0.0240 (5)	
C10	0.2804 (4)	0.1448 (3)	0.5328 (2)	0.0364 (6)	
H10A	0.4234	0.1391	0.5418	0.044*	
H10B	0.2237	0.0638	0.5659	0.044*	
C11	0.2075 (3)	0.1303 (2)	0.4108 (2)	0.0312 (5)	
C12	0.1880 (3)	0.2522 (2)	0.35072 (19)	0.0271 (5)	
C13	0.1380 (3)	0.2384 (3)	0.2367 (2)	0.0315 (5)	
H13	0.1258	0.3190	0.1975	0.038*	
C14	0.1064 (4)	0.1042 (3)	0.1821 (2)	0.0403 (6)	
H14	0.0743	0.0949	0.1060	0.048*	
C15	0.1224 (4)	−0.0156 (3)	0.2399 (2)	0.0437 (7)	
H15	0.0997	−0.1054	0.2028	0.052*	
C16	0.1723 (4)	−0.0027 (3)	0.3532 (2)	0.0395 (6)	
H16	0.1824	−0.0843	0.3914	0.047*	
N1	0.3885 (3)	0.8098 (2)	0.85102 (16)	0.0349 (5)	
H1N	0.4187	0.8619	0.9116	0.042*	
N2	0.2606 (3)	0.62466 (19)	0.45875 (14)	0.0249 (4)	
N3	0.2321 (3)	0.52067 (19)	0.37761 (14)	0.0248 (4)	
H3	0.2214	0.5357	0.3089	0.030*	
O1W	0.8185 (3)	0.6654 (3)	0.04112 (19)	0.0691 (7)	
H1WA	0.9413	0.6435	0.0647	0.083*	
H1WB	0.7964	0.5906	−0.0050	0.083*	
O2W	0.5429 (3)	0.85870 (19)	0.07832 (14)	0.0399 (5)	
H2WA	0.4837	0.7898	0.1118	0.048*	
H2WB	0.6532	0.8085	0.0732	0.048*	
Cl1	0.25495 (10)	0.60918 (7)	0.13519 (5)	0.0441 (2)	

### Atomic displacement parameters (Å^2^)

**Table:**

	*U*^11^	*U*^22^	*U*^33^	*U*^12^	*U*^13^	*U*^23^
C1	0.0428 (15)	0.0472 (17)	0.0268 (12)	0.0083 (12)	0.0023 (10)	0.0076 (11)
C2	0.0393 (14)	0.0321 (14)	0.0313 (12)	0.0048 (11)	0.0043 (10)	0.0078 (11)
C3	0.0193 (11)	0.0282 (13)	0.0277 (11)	0.0045 (9)	0.0045 (8)	0.0048 (9)
C4	0.0290 (12)	0.0281 (13)	0.0299 (12)	0.0040 (10)	0.0061 (9)	0.0043 (10)
C5	0.0298 (13)	0.0295 (13)	0.0362 (13)	0.0028 (10)	0.0062 (10)	−0.0008 (11)
C6	0.0206 (11)	0.0237 (12)	0.0259 (11)	0.0022 (9)	0.0043 (8)	0.0069 (9)
C7	0.0185 (11)	0.0239 (12)	0.0308 (11)	0.0033 (9)	0.0044 (9)	0.0057 (9)
C8	0.0338 (13)	0.0265 (13)	0.0383 (13)	0.0043 (10)	0.0067 (10)	0.0114 (11)
C9	0.0202 (11)	0.0216 (12)	0.0314 (12)	0.0028 (9)	0.0053 (9)	0.0062 (9)
C10	0.0356 (14)	0.0262 (14)	0.0503 (15)	0.0063 (10)	0.0094 (11)	0.0122 (12)
C11	0.0221 (12)	0.0234 (13)	0.0496 (14)	0.0024 (9)	0.0100 (10)	0.0042 (11)
C12	0.0200 (11)	0.0247 (13)	0.0372 (12)	0.0011 (9)	0.0080 (9)	0.0001 (10)
C13	0.0238 (12)	0.0305 (14)	0.0412 (13)	0.0021 (10)	0.0088 (10)	0.0005 (11)
C14	0.0342 (14)	0.0385 (16)	0.0463 (15)	−0.0001 (11)	0.0064 (11)	−0.0101 (13)
C15	0.0367 (15)	0.0264 (14)	0.0659 (18)	−0.0017 (11)	0.0103 (13)	−0.0144 (13)
C16	0.0321 (14)	0.0236 (13)	0.0637 (18)	0.0008 (10)	0.0104 (12)	0.0032 (12)
N1	0.0318 (11)	0.0424 (14)	0.0292 (10)	0.0034 (9)	0.0020 (8)	−0.0063 (9)
N2	0.0266 (10)	0.0214 (10)	0.0270 (9)	0.0023 (8)	0.0035 (7)	0.0033 (8)
N3	0.0286 (10)	0.0227 (10)	0.0232 (9)	0.0024 (8)	0.0030 (7)	0.0021 (8)
O1W	0.0588 (14)	0.0699 (16)	0.0761 (15)	0.0245 (12)	−0.0030 (11)	−0.0103 (12)
O2W	0.0382 (10)	0.0376 (11)	0.0409 (10)	0.0052 (8)	0.0003 (8)	−0.0142 (8)
Cl1	0.0570 (4)	0.0417 (4)	0.0343 (3)	0.0068 (3)	0.0048 (3)	0.0064 (3)

### Geometric parameters (Å, º)

**Table:**

C1—N1	1.330 (3)	C10—C11	1.514 (4)
C1—C2	1.378 (3)	C10—H10A	0.9700
C1—H1	0.9300	C10—H10B	0.9700
C2—C3	1.398 (3)	C11—C16	1.392 (3)
C2—H2	0.9300	C11—C12	1.413 (3)
C3—C4	1.395 (3)	C12—C13	1.395 (3)
C3—C6	1.464 (3)	C13—C14	1.385 (3)
C4—C5	1.365 (3)	C13—H13	0.9300
C4—H4	0.9300	C14—C15	1.378 (4)
C5—N1	1.344 (3)	C14—H14	0.9300
C5—H5	0.9300	C15—C16	1.386 (4)
C6—N2	1.353 (3)	C15—H15	0.9300
C6—C7	1.411 (3)	C16—H16	0.9300
C7—C9	1.380 (3)	N1—H1N	0.8600
C7—C8	1.507 (3)	N2—N3	1.339 (2)
C8—C10	1.532 (3)	N3—H3	0.8600
C8—H8A	0.9700	O1W—H1WA	0.8917
C8—H8B	0.9700	O1W—H1WB	0.8693
C9—N3	1.359 (3)	O2W—H2WA	0.8928
C9—C12	1.459 (3)	O2W—H2WB	0.9171
			
N1—C1—C2	120.6 (2)	C8—C10—H10A	108.1
N1—C1—H1	119.7	C11—C10—H10B	108.1
C2—C1—H1	119.7	C8—C10—H10B	108.1
C1—C2—C3	119.6 (2)	H10A—C10—H10B	107.3
C1—C2—H2	120.2	C16—C11—C12	118.1 (2)
C3—C2—H2	120.2	C16—C11—C10	121.2 (2)
C4—C3—C2	117.2 (2)	C12—C11—C10	120.5 (2)
C4—C3—C6	120.0 (2)	C13—C12—C11	120.4 (2)
C2—C3—C6	122.8 (2)	C13—C12—C9	123.8 (2)
C5—C4—C3	121.2 (2)	C11—C12—C9	115.8 (2)
C5—C4—H4	119.4	C14—C13—C12	119.8 (2)
C3—C4—H4	119.4	C14—C13—H13	120.1
N1—C5—C4	119.4 (2)	C12—C13—H13	120.1
N1—C5—H5	120.3	C15—C14—C13	120.4 (3)
C4—C5—H5	120.3	C15—C14—H14	119.8
N2—C6—C7	111.32 (19)	C13—C14—H14	119.8
N2—C6—C3	117.73 (19)	C14—C15—C16	120.2 (2)
C7—C6—C3	130.9 (2)	C14—C15—H15	119.9
C9—C7—C6	104.38 (19)	C16—C15—H15	119.9
C9—C7—C8	120.7 (2)	C15—C16—C11	121.1 (2)
C6—C7—C8	134.9 (2)	C15—C16—H16	119.4
C7—C8—C10	111.05 (19)	C11—C16—H16	119.4
C7—C8—H8A	109.4	C1—N1—C5	121.9 (2)
C10—C8—H8A	109.4	C1—N1—H1N	119.0
C7—C8—H8B	109.4	C5—N1—H1N	119.0
C10—C8—H8B	109.4	N3—N2—C6	104.52 (17)
H8A—C8—H8B	108.0	N2—N3—C9	112.78 (17)
N3—C9—C7	106.99 (19)	N2—N3—H3	123.6
N3—C9—C12	127.6 (2)	C9—N3—H3	123.6
C7—C9—C12	125.4 (2)	H1WA—O1W—H1WB	93.0
C11—C10—C8	116.6 (2)	H2WA—O2W—H2WB	92.1
C11—C10—H10A	108.1		
			
N1—C1—C2—C3	1.0 (4)	C16—C11—C12—C13	−1.0 (3)
C1—C2—C3—C4	−2.6 (3)	C10—C11—C12—C13	174.2 (2)
C1—C2—C3—C6	176.0 (2)	C16—C11—C12—C9	178.5 (2)
C2—C3—C4—C5	2.4 (3)	C10—C11—C12—C9	−6.2 (3)
C6—C3—C4—C5	−176.3 (2)	N3—C9—C12—C13	−7.7 (4)
C3—C4—C5—N1	−0.4 (3)	C7—C9—C12—C13	170.8 (2)
C4—C3—C6—N2	−0.9 (3)	N3—C9—C12—C11	172.8 (2)
C2—C3—C6—N2	−179.5 (2)	C7—C9—C12—C11	−8.8 (3)
C4—C3—C6—C7	176.5 (2)	C11—C12—C13—C14	0.3 (3)
C2—C3—C6—C7	−2.0 (4)	C9—C12—C13—C14	−179.2 (2)
N2—C6—C7—C9	−0.7 (2)	C12—C13—C14—C15	0.6 (4)
C3—C6—C7—C9	−178.3 (2)	C13—C14—C15—C16	−0.7 (4)
N2—C6—C7—C8	−177.6 (2)	C14—C15—C16—C11	−0.1 (4)
C3—C6—C7—C8	4.8 (4)	C12—C11—C16—C15	0.9 (3)
C9—C7—C8—C10	23.2 (3)	C10—C11—C16—C15	−174.3 (2)
C6—C7—C8—C10	−160.4 (2)	C2—C1—N1—C5	1.1 (4)
C6—C7—C9—N3	0.4 (2)	C4—C5—N1—C1	−1.4 (3)
C8—C7—C9—N3	177.81 (19)	C7—C6—N2—N3	0.7 (2)
C6—C7—C9—C12	−178.3 (2)	C3—C6—N2—N3	178.68 (17)
C8—C7—C9—C12	−0.9 (3)	C6—N2—N3—C9	−0.5 (2)
C7—C8—C10—C11	−36.3 (3)	C7—C9—N3—N2	0.0 (2)
C8—C10—C11—C16	−155.3 (2)	C12—C9—N3—N2	178.70 (19)
C8—C10—C11—C12	29.6 (3)		

### Hydrogen-bond geometry (Å, º)

**Table:**

*D*—H···*A*	*D*—H	H···*A*	*D*···*A*	*D*—H···*A*
N1—H1*N*···O2*W*^i^	0.86	2.12	2.856 (2)	143
N3—H3···Cl1	0.86	2.32	3.1618 (19)	167
O1*W*—H1*WA*···Cl1^ii^	0.89	2.25	3.142 (2)	174
O1*W*—H1*WB*···Cl1^iii^	0.87	2.37	3.237 (3)	178
O2*W*—H2*WA*···Cl1	0.89	2.29	3.139 (2)	158
O2*W*—H2*WB*···O1*W*	0.92	1.86	2.749 (3)	162

Symmetry codes: (i) *x*, *y*, *z*+1; (ii) *x*+1, *y*, *z*; (iii) −*x*+1, −*y*+1, −*z*.
